# Supramolecular Detoxification Approach of Endotoxin Through Host–Guest Complexation by a Giant Macrocycle

**DOI:** 10.3390/molecules30153188

**Published:** 2025-07-30

**Authors:** Junyi Chen, Xiang Yu, Shujie Lin, Zihan Fang, Shenghui Li, Liguo Xie, Zhibing Zheng, Qingbin Meng

**Affiliations:** Academy of Military Medical Sciences, Beijing 100850, China

**Keywords:** endotoxin, macrocycle, host–guest complexation, supramolecular detoxification approach

## Abstract

In Gram-negative bacteria, lipopolysaccharides (LPSs, also known as endotoxin) can induce extensive immune responses that will enable victims to produce severe septic shock syndrome. Because of the high mortality of sepsis in the face of standard treatment, advance detoxification schemes are urgently needed in clinics. Herein, we described a supramolecular detoxification approach via direct host–guest complexation by a giant macrocycle. Cationic pentaphen[3]arene (CPP3) bearing multiple quaternary ammonium groups was screened as a candidate antidote. CPP3 exhibited robust binding affinity toward LPS with an association constant of (4.79 ± 0.29) × 10^8^ M^−1^. Co-dosing with an equivalent amount of CPP3 has been demonstrated to decrease LPS-induced cytotoxicity on a cellular level through inhibiting ROS generation and proinflammatory cytokine expression. In vivo experiments have further proved that post-treatment by CPP3 could significantly improve the survival rate of LPS-poisoned mice from 0 to 100% over a period of 3 days, and inflammatory abnormalities and tissue damage were also alleviated.

## 1. Introduction

Endotoxin, a crucial pathogenic factor, is also known as a lipopolysaccharide (LPS) widely distributed in the cell wall of Gram-negative bacteria [1,2,3,4]. The chemical components of LPS mainly include three domains, namely lipid A, core oligosaccharide, and O-antigen polysaccharide [5,6,7,8]. When the bacterial cell lyses, LPSs, are released into the circulatory system and recognized by Toll-like receptor 4 of innate immune cells, this triggers the release of proinflammatory cytokines like tumor necrosis factor and interleukin-6, with the assistance of the lipopolysaccharides binding protein and leukocyte differentiation antigen 14 [9,10]. Hence the sepsis-associated signaling pathways are activated, sequentially causing diarrhea, fever, multiple organ failure, and even death [11,12,13]. According to rough statistics, the global number of annual LPS-induced sepsis cases exceeds 19 million, and its incidence rate has been increasing [14,15,16].

Currently, therapeutic schemes for LPS-induced sepsis mainly focus on infection control, hemodynamics maintenance, and major organ support [17,18,19]. Although research into LPS-associated sepsis has come a long way, there are still no specific reports from within the clinic. Antibiotics have been tried to treat sepsis; however, along with killing bacteria, more LPS is released, exacerbating the situation [20,21,22]. Analyzing its structure, toxicity primarily stems from negatively charged lipid A, which contains phosphorylated disaccharide and long-chain fatty acids [23,24,25,26]. To reach such a target, polymyxin B and many antibacterial peptides have been investigated to detoxify LPS via electrostatic charge neutralization. As this research topic develops in depth, researchers have found that the above agents can cause severe side effects, restricting their further clinical applications [27,28,29,30].

Alternatively, the supramolecular sequestration of toxins by synthetic receptors has been proven to be a practical approach to counteract their fatal virulence [31,32,33,34]. Such an approach works in a pharmacokinetic manner, seeking to reduce the concentration of free toxins or impede their interactions with organisms [35,36,37,38]. Sugammadex, a macrocyclic derivative, is the most successful case of the above sequestration strategy, which can reverse the muscle relaxation effect of rocuronium via specific molecule recognition [39]. However, strictly limited in the cavity capacity, traditional macrocycles are typically adept to encapsulate small- or medium-sized guests, rather than macromolecular toxins like LPSs [40,41,42,43]. The shape/size of extended biphen[n]arenes, a new family of macrocycles created by Li’s group, can be exactly customized by adjusting the structure of rigid monomers [44,45]. According to the structure of LPSs, we hypothesized that the anionic lipid A might be complexed by a positively charged giant macrocycle.

**Scheme 1:**
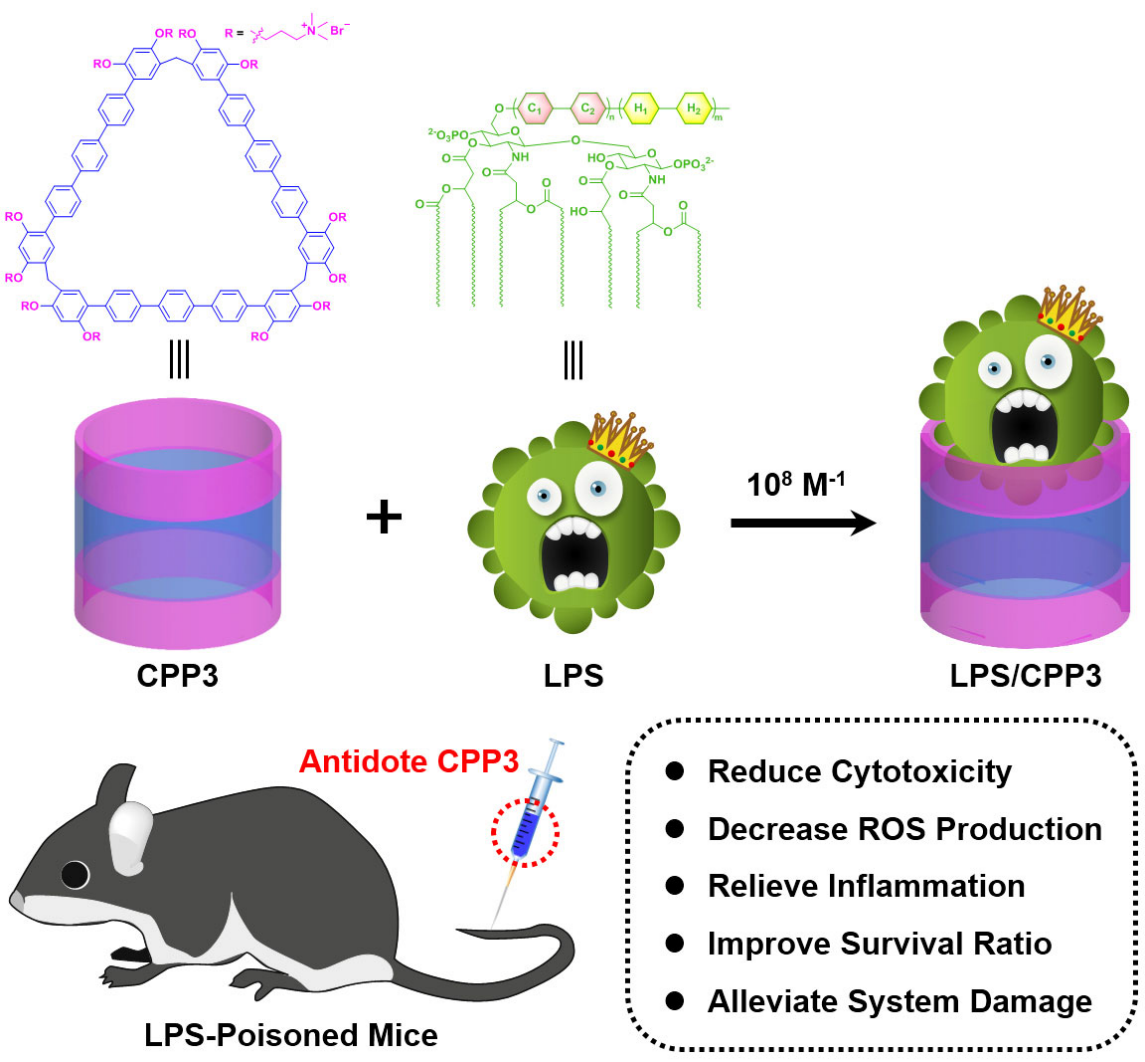
Chemical representations of cationic pentaphen[3]arene (CPP3) and lipopolysaccharides (LPSs), and schematic illustration of proposed supramolecular detoxification strategy via host–guest complexation by giant macrocycles.

Herein, we report an entire encapsulation approach to mitigate LPS-induced toxicity via host–guest interaction by macrocyclic antidote (Scheme 1). Quaternary ammonium-functionalized pentaphen[3]arene (CPP3) was screened out because of its ample cavity, fine biocompatibility, and robust recognition potency. Co-administration with an equivalent amount of CPP3 was able to inhibit LPS-induced toxicity on a cellar level via suppressing the generation of reactive oxygen species and the expression of proinflammatory cytokines. Moreover, post-treatment by CPP3 could also exert a detoxification function in vivo, manifesting the enhancement of the survival rate and relief of inflammation and tissue damage in LPS-poisoned model mice.

## 2. Results and Discussion

**Figure 1:**
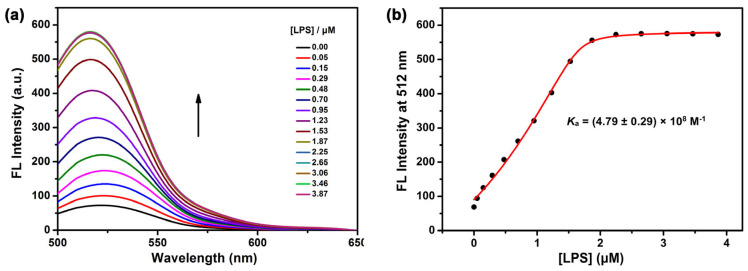
(**a**) Competitive fluorescence titration of LPSs in the presence of Fl (1.0 μM)/CPP3 (1.0 μM) in 10 mM HEPES buffer at pH = 7.4, *λ*_ex_ = 450 nm. (**b**) The associated competitive titration curve at *λ*_em_ = 513 nm and fitted *N*:1 competitive binding model. The *N* values were determined to be 0.60.

Using a fluorescent indicator displacement assay, the association constant (*K*_a_) between CPP3 and LPS was first quantitatively assessed with fluorescein (Fl) as the optimal reporter dye. Our previously reported work proved that FI with intense emission could be effectively quenched via the tight encapsulation of FI with CPP3′s cavity [46]. Upon the addition of LPS, for which the peak molecular weight was determined by a gel permeation chromatograph to calculate its concentration (Figure S1), the intrinsic emission of FI was recovered through competitive displacement (Figure 1a). As shown in Figure 1b, the change in the fluorescence intensity and concentration of LPS was fitted by an *N*:1 competitive binding model, giving a *K*_a_ value of (4.79 ± 0.29) × 10^8^ M^−1^ (*N* = 0.60). To further verify the complexation model, we utilized the fluorescent indicator displacement assay again under the same experimental conditions to measure the binding affinities of two negatively charged monosaccharide segments of LPS, α-D-glucosamine (GlcN) and 2-keto-3-deoxyoctonate (Kdo) with CPP3 (Figures S2 and S3). Upon the addition of these two monomer monosaccharides, no significant fluorescence regeneration of Fl was observed, indicating no complexation or at least quite weak interactions. That is to say that large-sized CPP3 was suited to entirely bind to LPS, but it could not afford the recognition to the anion monosaccharide region. In addition, a selectivity experiment for some biological molecules was conducted to exclude the possibility of competitive binding to CPP3. As shown in Figure S4, compared to LPSs, the addition of endogenous species, including adenosine phosphate, acidic amino acids, urea, glucose, etc., caused no obvious signal response, validating the good selectivity of CPP3 toward LPS.

**Figure 2:**
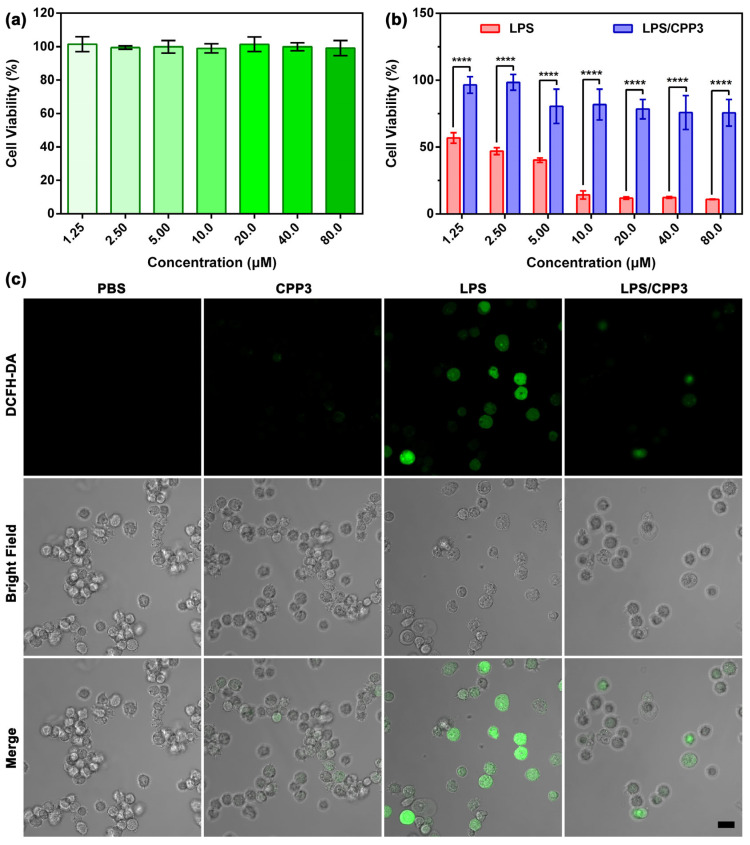
(**a**) Relative cell viability of RAW264.7 cells treated with different concentrations of CPP3 for 24 h (mean ± SD, n = 5). (**b**) Cytotoxicity seen in RAW264.7 cells after incubation with different concentrations of free LPS and LPS/CPP3 for 24 h (mean ± SD, n = 5). (**c**) Confocal laser scanning microscopy images of ROS production in RAW264.7 cells after administration with PBS, CPP3 (10 μM), LPS (10 μM), and that with or without CPP3. Scale bar = 20 μm. The significance of the difference in (**b**) was assessed using a *t*-test. **** *p* < 0.0001.

Previous studies have reported that LPSs were able to induce cells to produce reactive oxygen species (ROS) via multiple methods, leading to cell death [47,48]. In view of the above favorable data, we speculate that the complexation of LPS by CPP3 could impede the interaction between LPSs and cells, therefore relieving toxicity on a cellular level. To verify this hypothesis, cytotoxicity experiments were performed on a mouse monocyte macrophage leukemia (RAW264.7) cell line using a standard Cell Counting Kit-8 (CCK-8) assay. First, the cell viability of RAW264.7 cells treated with different concentrations of CPP3 was measured to remove interference. As shown in Figure 2a, CPP3 exhibited minimal cytotoxicity against RAW264.7 over the test range. For LPS, obvious toxicity could be observed, and even at a relatively low concentration (1.25 μM), the cell viability was only 56.80%. In sharp contrast, co-dosed with CPP3 was able to increase cell viability. For example, the cell viability increased from 11.00% to 75.66% at a concentration of 80.0 μM (Figure 2b).

**Figure 3:**
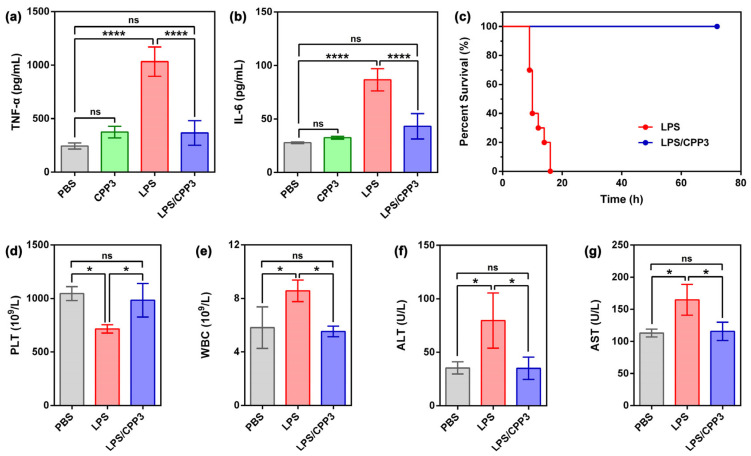
(**a**) TNF-α and (**b**) IL-6 levels in RAW264.7 cells after treatment with PBS, CPP3 (10 μM), LPS (10 μM), and LPS/CPP3 (10/10 μM) were assessed using the ELISA method (mean ± SD, n = 4). (**c**) Survival rate for 72 h of mice poisoned by intraperitoneal injection of LPS (15 mg∙kg^−1^), and one minute later, model mice were administrated with PBS and CPP3 (an equivalent dose of LPS) (n = 10 mice per group). (**d**) PLT, (**e**) WBC, (**f**) ALT, and (**g**) AST levels of mice treated by PBS, LPS (4.95 mg∙kg^−1^), and a mixture of LPS and CPP3 (an equivalent dose of LPS) for 3 days (mean ± SD, n = 3). Significance differences in (**a**,**b**,**d**–**g**) were assessed using one-way ANOVA tests and multiple comparisons. ns, not significant. * *p* < 0.05, **** *p* < 0.0001.

Next, the ROS production level of various scheme-treated cells was monitored by confocal laser scanning microscopy, and 2′,7′-dichlorodihydrofluorescein (DCFH) served as the fluorescent probe here. As shown in Figure 2c, the RAW264.7 cells administrated with PBS or CPP3 exhibited faint green fluorescence, which was ascribed to the physiological ROS in the cells. By comparison, bright green fluorescence was observed in the LPS group, revealing the sharp enhancement of ROS level. Under the same condition, co-administration with an equivalent amount of CPP3 could effectively suppress this evil consequence. As mentioned above, LPSs can be recognized by mononuclear phagocytes like RAW264.7, therefore increasing the secretion of cell factors like tumor necrosis factor (TNF-α) and interleukin-6 (IL-6). Next, we further verify the detoxification efficacy of CPP3 against LPS on RAW264.7 cells through measuring the cellular level of these two factors by an enzyme-linked immunosorbent assay kit. The appropriate calibration curves of TNF-α and IL-6 were first derived (Figures S5 and S6). As shown in Figure 3a,b, no significant change in these two factors was found in the RAW264.7 cells between the PBS group and CPP3 group. After treatment with 10 μM of LPS, the expression amounts of TNF-α and IL-6 increased by 3.23-fold and 2.19-fold, respectively, while co-dosing with CPP3 could relieve LPS-induced abnormalities to a normal level. Taken together, the above results led us to suggest that CPP3 could serve as a supramolecular antidote at the cellular level.

Our previous studies have preliminarily proved that CPP3 possesses good biocompatibility [46,47,48,49]. Given this, we would like to further verify whether CPP3 could exert detoxification potency in vivo. In present research, 15 mg∙kg^−1^ of LPS was intraperitoneally injected in Kunming mice to establish a poisoning model. Without the antidotal treatment of LPS intoxication, all of the animals died within 16 min (Figure 3c), demonstrating the lethal effect of this dose of inoculation. Remarkably, treatment with an equivalent amount of CPP3 at 1 min after poisoning significantly improved the survival rate, and 100% of the poisoned mice successfully remained alive over a course of 3 days.

**Figure 4:**
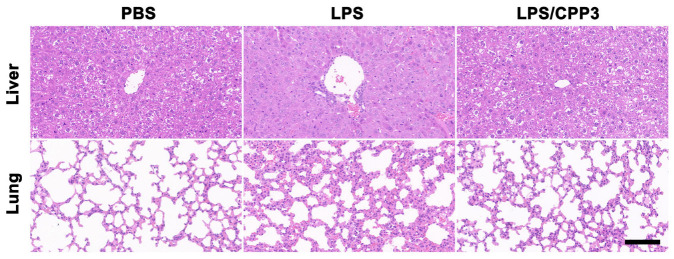
H&E-stained slice images of liver and lung from LPS (4.95 mg∙kg^−1^)-poisoned mice without antidotal treatment or administrated with CPP3 (an equivalent dose of LPS) for 3 days. Scale bar = 100 μm.

Further support of the excellent detoxification performance inferred for CPP3 was derived from hematological and histopathological analyses. A low dosage of LPS (4.95 mg∙kg^−1^) was used here to ensure the survival of model mice. After 3 days of treatment, all mice were euthanized, and blood samples and their liver and lung tissues were harvested. As shown in Figure 3d–g, abnormal levels of platelet (PLT, *p* < 0.05), white blood cells (WBCs, *p* < 0.05), alanine transaminase (ALT, *p* < 0.05), and aspartic transaminase (AST, *p* < 0.05) were obviously observed in LPS-poisoned mice. In contrast, administration with an equivalent amount of CPP3 could reverse the above index anomalies to their normal levels. Moreover, the morphological assessment of the liver and lung tissues of LPS-poisoned mice showed edema, hemorrhage, inflammatory infiltrates, and local necrosis, features that were characteristic of severe organ damage (Figure 4). For comparison, related symptomatic relief could be seen in the mice after the administration of CPP3. The above favorable findings revealed that CPP3 could serve as a supramolecular antidote in vivo against LPS intoxication.

## 3. Conclusions

In conclusion, we have proposed a feasible supramolecular detoxification approach that could suppress LPS-induced toxicity through direct host–guest complexation by a giant macrocycle. CPP3 exhibited effective binding potency toward LPS, rather than an anion monosaccharide unit with a *K*_a_ value in the vicinity of 10^8^ M^−1^. On the RAW264.7 cell line, co-dosing with CPP3 has been demonstrated to suppress LPS-induced cytotoxicity via decreasing ROS generation and proinflammatory cytokine expression. In vivo disintoxicating experiments further proved that post-treatment with an equivalent amount of CPP3 was able to improve the survival rate of LPS-poisoned model mice, alleviating related inflammatory abnormalities and tissue damage. Potentially, such a giant macrocycle could serve as a versatile supramolecular antidote toward macromolecular toxins like LPSs. Studies directly exploring this possibility are ongoing in our laboratory.

## Data Availability

The original contributions presented in this study are included in the article/Supplementary Materials. Further inquiries can be directed to the corresponding author(s).

## Supplementary Materials

The following supporting information can be downloaded at: https://www.mdpi.com/article/10.3390/molecules30153188/s1, Figure S1: Gel permeation chromatography of LPS, Figure S2: Competitive fluorescence titration of G1cN in the presence of FI and CPP3, Figure S3: Competitive fluorescence titration of Kdo in the presence of FI and CPP3, Figure S4: Fluorescence responses of FI/CPP3 towards LPS and some biological molecules, Figure S5: The standard curve of TNF-α by ELISA kit, Figure S6: The standard curve of IL-6 by ELISA kit [46,50].
