# Supplementary material for: Supramolecular Detoxification Approach of Endotoxin Through Host–Guest Complexation by a Giant Macrocycle
Source: Molecules. 2025 Jul 30;30(15):3188. doi: 10.3390/molecules30153188 (PMC12348354; doi:10.3390/molecules30153188)
Supplement: Supplementary file 1 [file molecules-30-03188-s001.zip › molecules-3782260-supplementary.pdf]

Supplementary Material

Supramolecular detoxification approach of endotoxin through host-guest complexation by a giant macrocycle

Junyi Chen, Xiang Yu, Shujie Lin, Zihan Fang, Shenghui Li, Liguo Xie, Zhibing Zheng* and Qingbin Meng*

Academy of Military Medical Sciences, Beijing 100850, P. R. China

* Corresponding authors.

Table of Contents

1 General materials and methods S3

1.1 Materials S3

1.2 Instruments S3

1.3 Cell and animals S3

1.4 Fluorescence titration S4

1.5 Cytotoxicity assay S4

1.6 Reactive oxygen species assay S4

1.7 Measurement of survival rate of LPS-poisoned mice S5

1.8 Detoxification efficacy *in vivo* S5

2 Supporting results and experimental raw data S6

2.1 Gel permeation chromatography of LPS S6

2.2 Binding of CPP3 with G1cN and Kdo S7

2.3 Fluorescence responses of FI/CPP3 towards LPS and some biological molecules S9

2.4 Standard curves of TNF-α and IL-6 levels by ELISA S10

References S12

1. General materials and methods

1.1 Materials

All the reagents and solvents were commercially available and used as received unless other specified purification. Cationic pentaphen[3]arene (CPP3) was synthesized according to a literature method.[1] Endotoxin (LPS, from *Eschericha coli* O111:B4), α-D-glucosamine (GlcN), 2-keto-3-deoxyoctonate (Kdo) and fluorescein (Fl) were purchased from Innochem (Beijing, China). Enzyme-linked immunosorbent assay (ELISA) kits for detection TNF-α and IL-6 were purchased from Raybiotech life, Inc. (Peachtree Corners, GA, USA). Roswell Park Memorial Institute 1640 medium was purchased from Gibco (Thermo Fisher Scientific). Fetal bovine serum (FBS), penicillin-streptomycin and PBS were purchased from Invitrogen (Carlsbad, CA, USA). The Cell Counting Kit-8 (CCK-8) was purchased from Dojindo China Co. Ltd. (Shanghai, China).

1.2 Instruments

Fluorescence spectroscopic studies were carried out using a FL-6500 fluorescence spectrophotometer, Perkin Elmer Co. Ltd. Cytotoxicity Assay was performed on Spectra Max®iD5 plate reader, Molecular Devices. The production of ROS was monitored by confocal laser scanning microscopy (LSM 510 META, Carl Zeiss, Germany).

1.3 Cell and animals

Mouse leukemia cells of monocyte macrophage (RAW264.7) cell line was purchased from the cell bank of Chinese Academy of Science. RAW264.7 cells were cultured in 1640 supplemented with 10% FBS, 1% penicillin and 1% streptomycin. Then cells were incubated at 37 °C under 5% CO_2 and 90% relative humidity, and passaged every 2 days.

Kunming mice (~20 g body weight) were purchased from the SPF Biotechnology Co. Ltd (Beijing) and maintained at 25 °C in a 12 h light/dark cycle with free access to food and water. Animals were allowed to acclimate to environment for at least one week before experiments. All experimental procedures were conducted in accordance with

the Guide for the Care and Use of Laboratory Animals of the AAALAC, and were approved by the Animal Care and Use Committee of the National Beijing Center for Drug Safety Evaluation and Research. Best efforts were made to minimize the number of animals used and their suffering.

1.4 Fluorescence titration

To quantitatively assess the binding affinity between CPP3 and LPS, fluorescence competitive titration was performed at 298 K in a 10 mM HEPES buffer at pH = 7.4 and FI was screened as an optical indicator. The experimental details were followed as the procedure reported previously. [2]

1.5 Cytotoxicity assay

The relative cytotoxicity of CPP3, LPS and LPS/CPP3 against RAW264.7 cells were assessed *in vitro* using CCK-8 according to the manufacturer's instructions. Cells were seeded into 96-well plates at a density of 8000 cells/well in 100 μL of 1640 supplemented with 10% FBS, 1% penicillin, and 1% streptomycin and cultured for 24 h in 5% CO_2 at 37 °C. The culture medium was replaced with 100 μL of a fresh medium containing various concentrations of CPP3 (1.25, 2.5, 5, 10, 20, 40, and 80 μM), LPS (1.25, 2.5, 5, 10, 20, 40, and 80 μM) or LPS/CPP3 (1.25, 2.5, 5, 10, 20, 40, and 80 μM) and were further incubated at 37 °C for 24 h. Note: CPP3 could be fully soluble in aqueous buffer within the concentration of 80 μM at least. Subsequently, 100 μL of CCK-8 was added into each well and incubated for another 0.5 h in the dark condition. The plates were then measured at 450 nm using a plate reader. All experiments were carried out five independent times. Cell viability was calculated as follows:

$$\text{Cell Viability} = \frac{\text{OD}_{\text{test}} - \text{OD}_{\text{blank}}}{\text{OD}_{\text{control}} - \text{OD}_{\text{blank}}} \times 100\%$$

Where OD_{blank} is the optical density of blank well (medium and CCK-8 reagent), OD_{test} is the optical density of the test group and $OD_{control}$ is the optical density of the control group.

1.6 Reactive oxygen species assay

Reactive oxygen species (ROS) generation was assessed *in vitro* using RAW264.7 cells. After treated with PBS, CPP3 (10 μM), LPS (10 μM) and LPS/CPP3 (10 μM/10

μM) for 24 h, 10 μL of DCFH-DA (10 μM) was added and incubated for 30 min, followed by PBS washing. The production of ROS were monitored by confocal laser scanning microscopy.

1.7 Measurement of survival rate of LPS-poisoned mice

Twenty kunming mice were randomly divided into 2 groups. A 200 μL of PBS or CPP3 (9.91 $mg{\cdot}kg^{-1}$, an equivalent dose of LPS) were intraperitoneally administrated in mice at 1 min after LPS (15.00 $mg{\cdot}kg^{-1}$) poisoning. Above mice were observed for 3 days and the mortality rate of each group was recorded.

1.8 Detoxification efficacy *in vivo*

For routine blood, blood biochemistry and H&E stainning, 9 mice were randomly divided into 3 groups. PBS, LPS (4.95 $mg{\cdot}kg^{-1}$) and LPS/CPP3 (4.95/3.27 $mg{\cdot}kg^{-1}$) were injected intraperitoneally. After 3 days treatment, the mice were euthanized, and blood samples were collected for hematological analysis. In addition, histopathological sections of liver and lung were prepared and stained with hematoxylin and eosin, followed by imaging with optical microscopy.

2. Supporting results and experimental raw data

2.1 Gel permeation chromatography of LPS

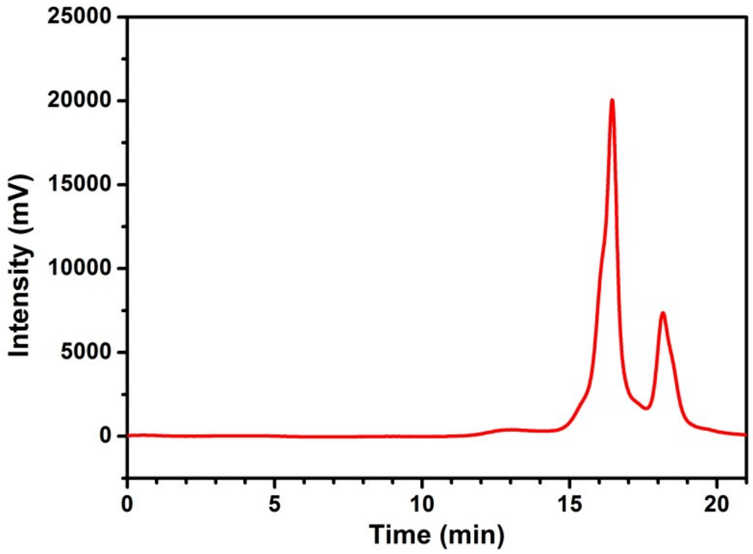

Figure S1. The peak molecular weight of LPS was determined to be 5353 by gel permeation chromatography.

2.2 Binding of CPP3 with G1cN and Kdo

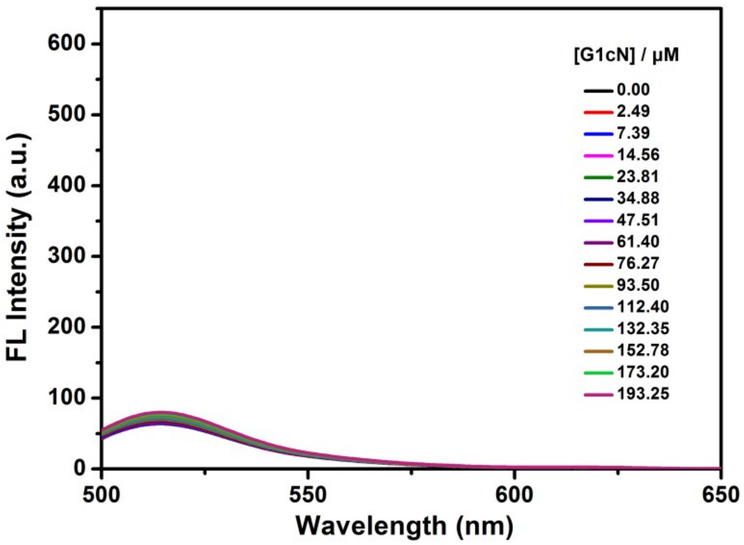

Figure S2. Competitive fluorescence titration of G1cN in the presence of FI (1.00 μM) and the CPP3 (1.00 μM) in 10 mM HEPES buffer at pH 7.4, λ_{ex} = 450 nm. The recovery of fluorescence is too small relative to the range of quenching, so no reasonable association constant can be obtained.

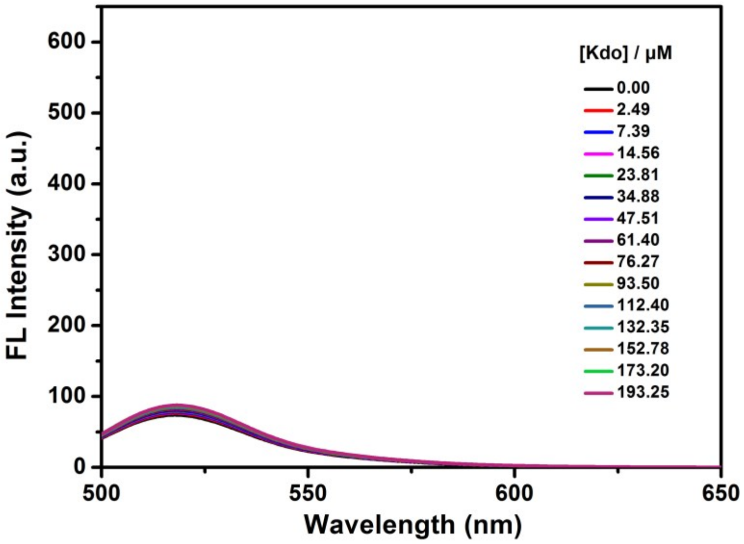

Figure S3. Competitive fluorescence titration of Kdo in the presence of FI (1.00 μM) and the CPP3 (1.00 μM) in 10 mM HEPES buffer at pH 7.4, λ_{ex} = 450 nm. The recovery of fluorescence is too small relative to the range of quenching, so no reasonable association constant can be obtained.

2.3 Fluorescence responses of FI/CPP3 towards LPS and some biological molecules

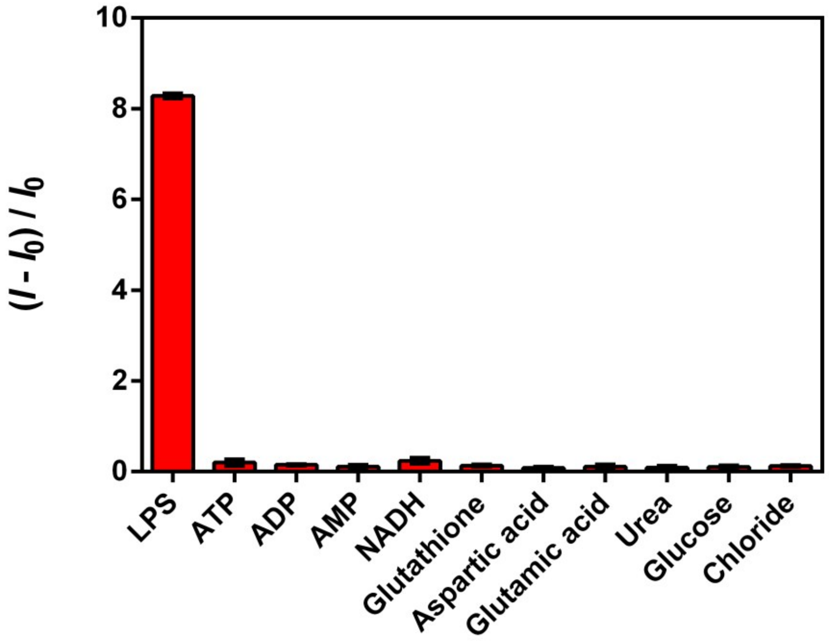

Figure S4. Fluorescence responses of Fl (1.0 μM)/CPP3 (1.0 μM) at 513 nm (λ_{ex} = 450 nm) upon addition of LPS and some representative biological co-existing species (10 μM) in HEPES buffer. Data were from n = 3 independent experiments and are presented as mean ± SD.

2.4 Standard curves of TNF-α and IL-6 levels by ELISA

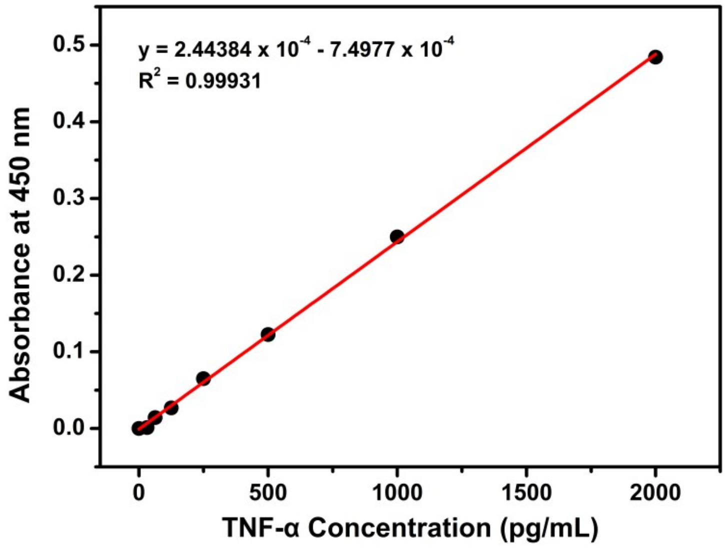

Figure S5. The standard curve of TNF-α by ELISA kit.

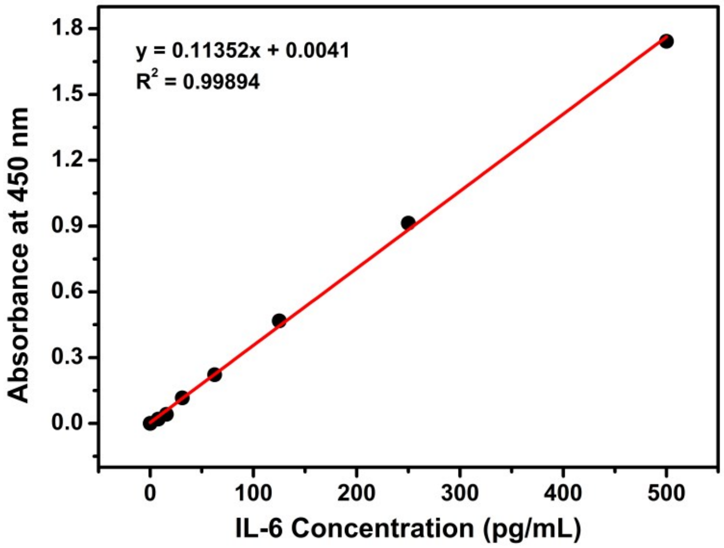

Figure S6. The standard curve of IL-6 by ELISA kit.

References

1. X. Yu, Y.-H. Zhang, L. Tian, F. Zhang, Z.-L. Zhang, L.-M. Chen, J.-Y. Chen, C.-J. Li and Q.-B. Meng, *Cell Rep. Phys. Sci.*, **2024**, *5*, 102044.
2. A. Hennig, H. Bakirci, W. M. Nau, *Nat. Methods*, **2007**, *4*, 629.
